# Informing the delivery of cancer survivorship care in rural primary care practice

**DOI:** 10.1007/s11764-021-01134-3

**Published:** 2022-02-02

**Authors:** J. R. Klemp, C. J. Knight, B. Lowry, T. Long, C. Bush, K. Alsman, H. Krebill, D. Peereboom, L. Overholser, K. A. Greiner

**Affiliations:** 1 University of Kansas Cancer Center, University of Kansas Medical Center, Kansas City, KS USA; 2 Department of Medicine, University of Kansas Medical Center, Kansas City, KS USA; 3 Masonic Cancer Alliance, The University of Kansas Cancer Center, Kansas City, KS USA; 4 Ascension, Wichita, KS USA; 5 Johns Hopkins Bloomberg School of Public Health, Baltimore, MD USA; 6 University of Colorado School of Medicine, Aurora, CO USA; 7 Department of Family Medicine, University of Kansas Medical Center, Kansas City, KS USA

**Keywords:** Cancer survivorship, Rural primary care

## Abstract

**Purpose:**

The cancer survivor population is projected to increase to 22.2 million by 2030, requiring improved collaboration between oncology and primary care practices (PCP). PCPs may feel ill-equipped to provide cancer survivorship care to patients without input from cancer specialists. Compared with nonrural cancer survivors, rural cancer survivors report experiencing worse treatment-related symptoms. The goal of this study was to gain a better understanding of the perspectives of PCP teams towards survivorship care and to develop and test an interdisciplinary training program to improve cancer survivorship care in rural practice.

**Methods:**

This study was conducted in two phases. First, focus groups were conducted with rural PCP teams to gather information regarding beliefs, practices, and barriers related to cancer survivorship care delivery. A thematic analysis was completed using an iterative process of reviewing transcripts. Results from phase 1 were used to inform the development of a pilot intervention tested within seven rural PCPs (phase 2). Pre- and post-intervention knowledge changes were compared, and post-session interviews assessed planned or sustained practice changes.

**Results:**

Seven PCPs participated in focus groups (phase 1). Cross-cutting themes identified included (1) organizational barriers affecting the delivery of cancer survivorship care, (2) challenges of role delineation with specialists and patients, (3) difficulty accessing survivorship care and resources, and (4) providers’ lack of knowledge of cancer survivorship care. For phase 2, seven practices participated in four case-based educational sessions. Within and between practice changes were identified.

**Conclusion:**

This project explored cancer survivorship perspectives among PCP teams. Lack of familiarity with evidence-based guidelines and the inability to identify cancer survivors was apparent during discussions and led to the implementation of the phase 2 intervention, iSurvive. As a result, PCPs either changed or planned changes to improve the identification and evidence-based care of cancer survivors.

**Implications for Cancer Survivors:**

Address barriers to access cancer survivorship care in rural primary care practices.

## Introduction

While the incidence and mortality from cancer in the US continue to decline, the morbidity associated with treatment and recovery is a growing concern [1]. The total number of cancer survivors is expected to increase for decades to come. It is projected that by 2030 there will be over 22.2 million cancer survivors [2]. This will place a great burden on the oncology care workforce and require improved coordination and collaboration between oncology and primary care providers (PCP). Even at present, cancer survivorship care remains inadequate for the vast majority of patients who have decreased the frequency or stopped seeing their oncologists and returned to routine care with a PCP [3, 4]. Significant research has documented that these PCPs may feel ill equipped to provide guideline-concordant cancer survivorship care to patients without input from cancer specialists [4, 5]. Unfortunately, with the rising volume of cancer survivors, it is unlikely that oncology providers can indefinitely see these patients once in remission or during extended therapy. Therefore, it is crucial to prepare PCPs to deliver evidence-based and guideline concordant survivorship care.

Cancer survivorship care requires versatility and flexibility to meet the needs of the survivor population in diverse care settings [6]. There has been little research on the impact of survivorship care plans on rural survivors, however, when compared with nonrural cancer survivors, rural cancer survivors report experiencing worse treatment-related physical and psychological symptoms [7–9]. Rural survivors, who may live many miles from large hospitals and cancer treatment facilities, face unique challenges to receiving guideline appropriate cancer survivorship care [10]. They may stop seeing oncologists and return to PCPs sooner than others. Travel and costs may lead them to disengage from their cancer providers sooner than those residing in urban areas. These factors may also limit their ability to receive the additional testing and services that survivorship guidelines recommend. There is some evidence that rural survivors favor receiving management for complex conditions from their local, rural-based PCPs [11]. Therefore, a greater understanding of the barriers to improve access and the delivery of evidence-based care is needed.

Most reports describing the management of cancer survivors in primary care have relied on the qualitative survey and qualitative studies [4]. While some qualitative studies and quality improvement projects have been conducted with PCPs, few have focused on rural PCPs or rural cancer survivorship care. In addition, some qualitative studies have evaluated different types of survivorship care plan formats [12]. Many of these were conducted outside of the USA [12–15], and have focused exclusively on providers, particularly the physician provider, rather than the primary care team [13–19].

The goal of this two-phase pilot study was the following:

- Phase 1: Gain a better understanding of the perspectives of primary care teams towards survivorship care. We explored knowledge, current practices, perceived barriers to improvement, and resource needs among rural PCP teams. By using structured and open-ended questions and various prompts, we attempted to elucidate the opinions of not just providers but nurses, medical assistants, administrative staff, and others.
- Phase 2: Test iSurvive, a 4-session, in-person, case-based, curriculum focused on common cancers, late and long-term effects of cancer, and practice facilitation aimed at improving the identification and management of cancer survivors in the practice.

## Methods

### Study setting

**Fig. 1:**
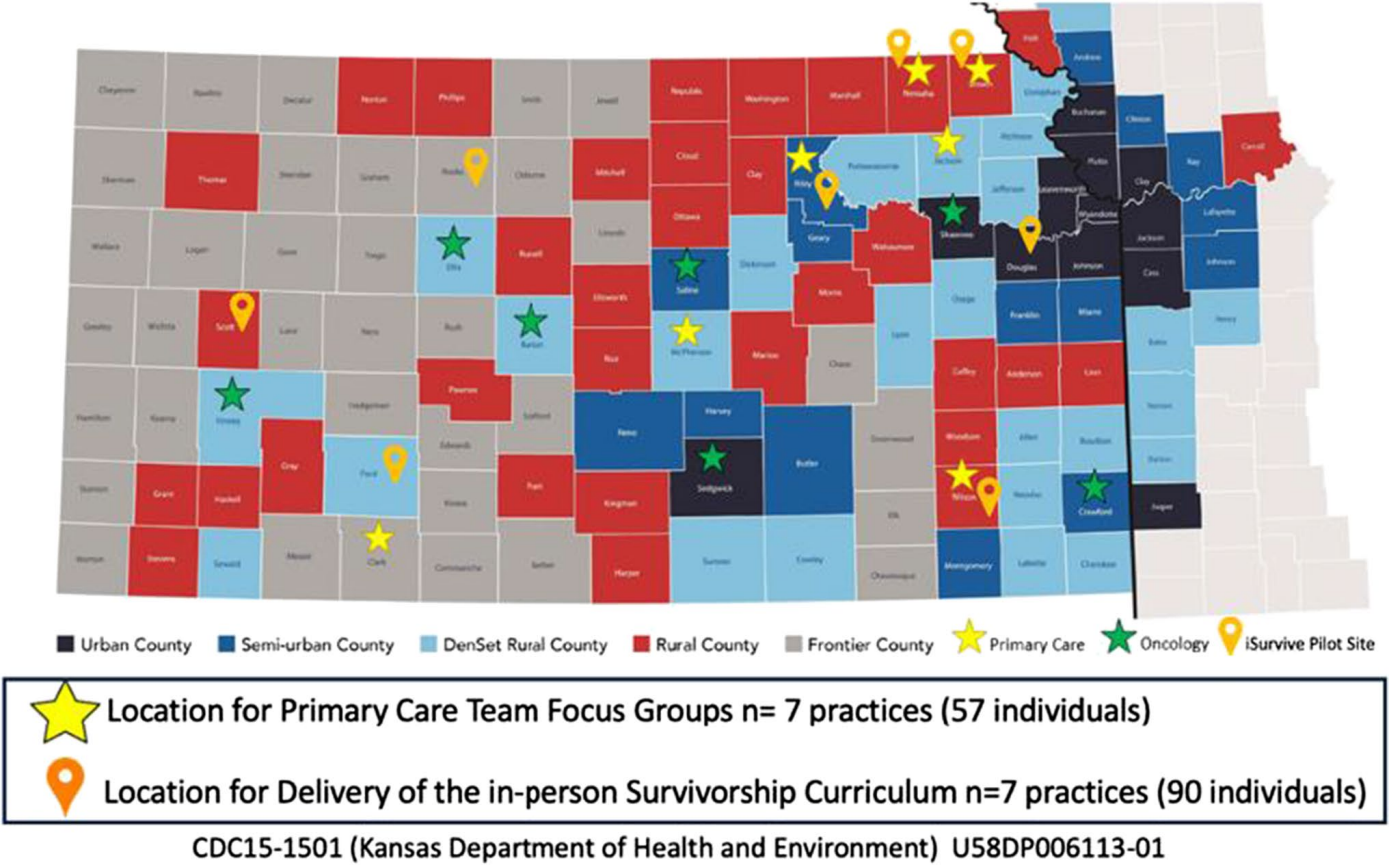
Rural PCP Practices Participating in Phase 1 & Phase 2

The University of Kansas Cancer Center (KUCC), a National Cancer Institute-designated Cancer Center, is the only academically based clinical and basic research center in the region. Located in the Kansas City metro area, the KUCC has a catchment area of 123 counties and 4.5 million residents that represent the state of Kansas and Western Missouri (Fig. 1). Of the 123 counties in the catchment area, 78% (*N* = 96) are classified as rural based on the Rural–Urban Continuum Codes (RUCC). Additionally, of the 4.5 million residents located in the 123 counties, 25% of the population are rural. Its unique location and residents make it an ideal setting for this study.

The KUCC’s outreach network, the Masonic Cancer Alliance (MCA), collaborates with and supports a primary care, practice-based research network, the Kansas Patients and Providers Engaged in Prevention Research (KPPEPR), of which a vast majority also participate in medical student and resident education (https://www.masoniccanceralliance.org/kppepr.html). KPPEPR covers KUCC’s 123 county catchment area and has a membership of over 60 rural PCPs that have participated in research projects and activities over the last 16 years. Members include physicians, behavioral specialists, advanced practice providers, nurse navigators, outreach, and research coordinators. In general, the MCA represents a network of oncologists while KPPEPR represents a network of primary care practices and providers.

#### Study design

This study was conducted in two phases. In phase 1, qualitative formative methods were used to obtain in-depth information regarding primary care team beliefs, current practices, and barriers related to cancer survivorship care. This data was then analyzed and used to the guide development of a pilot training intervention that was tailored to the regional landscape and tested within seven rural primary care practices (phase 2).

##### Phase 1

In order to get in-depth and well-rounded information from various perspectives within a practice, particularly around barriers to optimal care and coordination, focus groups were designed to include all members of the practice team rather than only providers. All focus groups were conducted from September to November 2017. The study was reviewed and approved by the University of Kansas Medical Center Institutional Review Board prior to participant recruitment.

##### Recruitment

An invitational email was sent out to rural PCPs within the KPPEPR network for voluntary participation. Practices that showed interest were contacted by the study team. A total of seven practices and 57 individuals were recruited as depicted by yellow stars in Fig. 1. There was no practice incentive, but lunch was provided to participants to thank them for their time.

##### Setting

Each focus group, lasting up to an hour, was conducted in-person at the participating rural practice site, with the exception of one group which was conducted remotely by Zoom videoconferencing due to scheduling. Requirements for participation included having at least three members of the practice involved in each focus group, with at least one member being a physician, in addition to advanced practice providers, nursing staff, medical assistants, and administrative staff, in order to explore the roles and views that all team members might have regarding patient care and practice flow, and more specifically, delivery of cancer survivorship care. Three trained team facilitators worked together to lead the discussion during each focus group. The lead facilitator was a primary care physician and co-facilitators asked questions as needed or assisted with clarifying questions.

##### Instruments

Verbal consent was obtained at the beginning of each focus group from all participants. An interview guide and script were developed to highlight primary study questions and to organize the sequence of topics addressed within these group conversations. Within the guide, we explored knowledge, current practices, perceived barriers to improvement, and resource needs among the entire interdisciplinary rural practice team. We used open-ended questions and various prompts in an attempt to elucidate the opinions of not just providers but nurses, medical assistants, administrative staff and others. Because cancer survivorship care and improvement may not be familiar to primary care teams, the initial focus group questions were structured to spark discussion about chronic disease management. An appreciative inquiry approach was used to help the practice team brainstorm the ways in which they conduct chronic disease management at their practice and how they could apply existing quality improvement systems, supports and established roles to cancer survivorship care. Facilitators allowed participants considerable latitude for self-direction within this framework to explore issues and barriers faced by their practice. Facilitators distributed examples of Survivorship Care Plan (SCP) templates during each focus group. Participants were asked whether they had ever received similar documents from oncologists and what they liked or did not like about the SCPs. In addition, facilitators asked for feedback on a patient survivorship manual developed by practices and community members in rural Colorado [20]. The manual was developed to foster education, promote guidelines for survivorship care, and delineate roles between survivors and their various medical providers and teams.

##### Data analysis

Focus groups were audio recorded and transcribed by an experienced transcriptionist. Qualitative analysis was conducted using an iterative process and following the techniques of Miles and Huberman [21]. Transcripts were reviewed, and themes were identified. Triangulation and consensus were used throughout the analysis phase to maximize the reliability and validity of the process and results.

##### Phase 2

**Recruitment and Setting** Seven practices (4 practices were part of phase 1) were recruited for participation in phase 2 as indicated by the orange pins in Fig. 1. Case-based educational sessions followed a lunch-and-learn format. Two outreach facilitators provided four separate, in-person 1-h sessions at the practice location. Facilitators were trained by a nurse educator who had experience conducting similar sessions in rural Colorado primary care practices as part of the “iSurvive” survivorship education program [20]. Both facilitators had extensive educational and health promotion experience, one being a physician assistant and the other a cancer nurse navigator. All practice staff were invited to these sessions.

##### Curriculum

Content for the sessions was adapted by our research team (Klemp, Lowry, and Alsman) and advocates with permission from the iSurvive survivorship educational program [20]. Presentations included evidence-based cancer survivorship care guidelines, case examples for common cancers and effects of cancer, strategies for changing practice workflows, and session-specific resources. The content was also tailored to the local and regional context and each session allowed for interaction, discussion, and questions. Due to limited space in the practice sites, slide presentations were often projected onto a wall, and for the majority of practices, pre-printed handouts of the slides were distributed to participants. All sites preferred to have paper handouts for notetaking and reference material.

##### Instruments

Verbal and pen/paper surveys were administered to all attendees of the lunch-and-learn sessions. Basic assessments on knowledge of cancer survivorship care were administered prior to session one and after session four. Finally, practices were recontacted 3–6 months after they had completed their 4th session to complete a brief telephone interview to assess any practice changes or sustained alterations in the delivery of cancer survivorship care for their patients.

##### Data analysis

Survey data was compiled in a REDCap database and basic distributions and frequencies were calculated. Pre- and post-cancer survivorship knowledge was scored and summed responses were compared. Cancer survivorship knowledge changes from pre to post were compared using a paired sample Student’s *t*-test. Post-session interviews were also used to assess any sustained practice changes.

### Study results

#### Phase 1

Of the 17 practices contacted, 12 responded to either accept, decline, or ask further questions about the project, and eight practices agreed to participate in the focus groups. One practice was excluded from the results because only one member of the practice participated. Table 1 shows practice characteristics for the seven practices that participated in focus groups. Practices were located across Kansas (KS) (Fig. 1). Rurality of practice location based on Kansas Department of Health and Environment classifications of population density, ranged from semi-urban to frontier: 1 semi-urban, 2 densely settled rural, 3 rural, and 1 frontier (22). The average number of providers, physicians, and advanced practice providers (APPs), in each practice, was eight. Five out of seven practices were hospital-owned, and three were members of an Accountable Care Organization (ACO). Almost all (6/7) of the practices were using an electronic health record (EHR): Cerner, eClinicalWorks, and CPSI.

**Table 1:** Primary care practice characteristics

Location in KS	Level rurality	Total # providers*	Practice ownership	ACO
Southeast	Rural	7	Independent	Yes
Southwest	Frontier	7	Hospital-owned	No
Central	Semi-urban	13	Independent	
Northeast	Rural	9	Hospital owned	Yes
Northeast	Rural	6	Hospital owned	No
Central	Densely settled rural	7	Hospital owned	No
Northeast	Densely settled rural	8	Hospital owned	Yes

^*^Providers, physicians and advanced practice providers (PA or NP)

### Cross-cutting themes

The team identified four cross-cutting themes related to cancer survivorship care in primary care practices. Although themes were individual in nature, there were aspects that overlapped and were separated into sub-themes outlined in Table 2 [22].

**Table 2:** Themes related to cancer survivorship in primary care practices

Cross-cutting themes	Summary statements from primary care interviews	Participant quotes from primary care interviews
Organizational structure	•Broader healthcare issue • Lack of EHR integration and systems within practice: cannot identify survivors • Difficulty finding and consolidating information from various sources: ***scanned documents are essentially worthless. If it is not on the problem list, it is not on the providers radar*** • Practices participating in ACOs appeared more equipped to support care coordination	*• “Its one thing to collect the data, it’s another thing to input the data and then it's another thing to put the data in the right place where you can come back and retrieve it in a systematic format. So you have three areas then that have the potential for breakdown or challenges.*^*”*^ *•”Have a more organized approach to what we do would be very helpful. yes. But that requires the whole system to change, not just one part of it:* • “So *a single EMR that everybody uses that was the same format…that would change medicine.”*
Provider and patient engagement and communication	• Broader healthcare issue • PCP's want 1–2 pages with specific recommendations. PCPs still see oncologists as the experts who should be providing recommendations • PCPs mentioned the different approaches to transitioning to palliative care. Oncology wants to • PCPs mentioned the different approaches to transitioning to palliative care. Oncology wants to continue treatment. PCPs are concerned about the negative effects of the treatment on the patient and their family • No consensus on how much responsibility patients should have. Some wanted the patient to have more responsibility, but others felt like patients were not reliable • Rural: personal relationships. better communication with patients and specialists than in urban communities	•”There's nothing more confusing to the patient than we tell them three years and the oncologist says seven. the internet says don't take it at all… so if we had a way to know what the oncologist was thinking." • “*Well pick oncologists that have an excellent coordinator. I won't go to an oncologist or send someone to someone that doesn't have a good coordinator to help me. We use doctors who either call us directly themselves or they have someone that contacts us. When they need help. they don't leave us in the dark."* • *"****And then you get like 432 pages of notes that you have to try to sift through and figure out where everything is****."* •”*We do know them (specialists) very personally. We will go out to dinner with them when they are here… when I have a diagnosis of cancer, I will text them right away and say can you see them and they'll say yup… We do have those good relationships.*^*”*^
Access to survivorship care and resources	Lack of survivorship resources in rural communities. particularly with mental health support	•”*I don't know of any community that has good mental health, not in Kansas. We have probably the poorest mental health there is."* •”*A lot of patients can't go see a specialist because they can't afford to go or they don't have the transportation means. We have very limited resources out here.”*
Knowledge gaps	PCP teams shifted the conversation to acute cancer treatment issues (transportation, fundraisers for treatment), rather than long-term survivorship issues	• *“I always just assume that I'm letting the oncologist figure that stuff out. but I am not real well-educated on all those things I should be doing 5. 20.* 15 years* later. For sure, I think there's a gap in knowledge base for more physicians."*

#### Phase 2

Lunch-and-learn sessions with each of the seven practices were conveniently scheduled. Practices learned to identify resources available to help both providers and cancer survivors in primary care practices in rural Kansas. Knowledge about surveillance needs and common physical and emotional late and long-term effects experienced in the treatment of cancer were reviewed and team-based strategies to help meet the needs of cancer survivors in their practices were discussed and developed. Each practice was given a verbal “pretest” asking practices to answer as a group, questions about confidence in identifying resources and guidelines for cancer survivors in their practice, how they identify patients who are cancer survivors (answers using Likert scale), what challenges they see within their clinic in addressing the needs of cancer survivors, and if they receive a cancer survivorship care plan from their cancer patients. The practices were also asked what their objectives were for this training.

Participants in these sessions included physicians (*n* = 17); advanced practice providers (*n* = 9); administration staff (*n* = 64). In a final session survey, all seven practice sites and all participants characterized the curriculum as valuable and reported that they had learned new information they planned to use in their practice.

Sessions were knowledge based with interactive discussions around the cases presented. Participants were asked how their practices would handle each case and what challenges might be encountered including survivorship within a chronic disease framework, risk-stratified survivorship care (i.e., cardio-toxicity, cancer genetics, etc.), guideline-concordant cancer screening guidelines, and identification of late and long-term effects of cancer [23]. At the end of each curriculum session, the practice participants were instructed to think about how they could apply what was learned during the training session to their existing chronic care practice models. A “thinking points” sheet/homework was handed out to jot down thoughts about the following:

1. How caring for an individual with a history of cancer is similar to caring for individuals with other chronic conditions? How is it different?
2. What assets could you utilize in the practice setting to help ensure cancer survivors are receiving the care and support they need?

Practice pre and posttests were administered and compared between sites. Responses showed that PCP teams rarely or never received a survivorship care plan, routinely did not have a formal process to identify cancer survivors within their practice, were unaware of where to access resources for cancer survivors and were unclear which cancer screening guidelines to follow.

Each practice was asked to suggest additional training topics for future initiatives or a wish list of additional training materials. Results are summarized in Table 3.

**Table 3:** Wish list of additional training material topics and resources

Care coordination between primary care providers and oncology specialists and explicit role delineation to determine who is doing what for follow-up care
Health promotion in PCP offices such as smoking cessation, nutritional health and physical activity for cancer survivors
Financial toxicity for cancer patients
Narrow the communication gap between PCPs and oncologists
Receive a survivorship care plan from oncologists
Additional mental health and counseling resources
Additional social support for cancer patients in the community
Pocket cards/posters for the clinic with current NCCN guidelines for screening and risk assessment

#### Six-month follow-up phone interviews

Approximately 3–6 months after completing the iSurvive curriculum, follow-up calls were made to all seven sites with responses obtained from six sites. Questions asked included, “What changes did you make in your practice to address the needs of cancer survivors?”, “How did you go about making those changes?”, “What barriers have you faced since the training implementing the survivorship guidelines?”, and “What information in the training was helpful for you and the practice?”.

Practices reported having more awareness of cancer survivors and prioritizing the management of late and long-term effects of cancer. All noted that the goal of improving the identification of cancer survivors within their practice and following risk stratification guidelines remained a challenge.

## Discussion

This two-phase pilot project was undertaken to better understand the challenges rural primary care teams face when managing cancer survivors. To date, there has been little work done to address the unmet needs of rural cancer survivors and the unique challenges that primary care providers face in the delivery of care to cancer survivors.

Rural primary care practices have limited capacity and few incentives to implement guideline-concordant cancer survivorship care. It is also unclear whether practices are aware of survivorship care deficiencies and whether they have received survivorship care plans (treatment summary and follow-up recommendations) from oncology providers. Change in primary care practice usually results when common problems are tied to a quality measure accompanied by performance incentives [24]. Survivorship care may not seem like a common problem and has difficult to measure quality indicators. For most primary care practices, survivorship care is not at the top of the list of competing priorities [25]. Efforts must be made to improve quality metrics in cancer survivorship care and building this framework is vital to the sustainability and scability of delivering high-quality survivorship care [23]. One useful metric would be a measure of how well PCPs identify cancer survivors in the practice. Another would be a clear measure of delivery of guideline-concordant screening services. Such a measure would need to be tailored to the survivor’s cancer type, cancer treatment received, and other risk factors. More research in this area is needed to move the bar forward and create clear targets for PCP improvement. Results from phase 1 highlight the cross-cutting issues experienced by primary care teams including organizational structure, provider and patient engagement and communication, access to survivorship care and resources, and knowledge gaps [22]. Practices in phase 1 recognized the need to identify cancer survivors in their practice but felt frustrated by the lack of a formal or systematic solution.

To address these issues, we implemented phase 2 of this pilot project. Attempting to build on the limited research undertaken in this area, we incorporated similar, yet updated methods, used by Overholser and colleagues in the iSurvive Program [20]. Learning from our colleagues and developing an updated curriculum informed by phase 1 of our pilot, both projects received favorable reviews from participants who planned to put this new or refined knowledge into practice. However, after a short period of time (3–6 months) the practices in phase 2 of our pilot continued to experience challenges implementing change.

We identified the next steps, from phase 1 and phase 2 of the pilot: in order to foster practice change, there would need to be a more formal approach including practice facilitation to help practices identify goals, utilize current staff, update workflows (algorithms), incorporate risk stratification, etc., and ultimately, develop EHR order sets that could be implemented to make the management of cancer survivors more systematic [26].

### Limitations

This pilot work was limited to primarily qualitative data collection, which was influenced by the current burden put upon rural primary care practices. With numerous competing priorities and limited resources, our study team was mindful of the data collection process and gleaned as much from the qualitative interviews as possible. This tactic was well-received by the practices and thus provided an enriched understanding of the barriers and opportunities within rural primary care practices.

## Conclusion

Primary care teams face similar cross-cutting issues in caring for cancer survivors including educational gaps, communication of history, treatment and recommendations, EHR integration, and lack of resources. Based on the complex needs of cancer survivors and the complexity of health care delivery, an organized approach is needed to align cancer survivorship care delivery across settings. There is an opportunity to test educational models and implementation strategies in a variety of healthcare delivery settings in order to increase knowledge and practice of survivorship care among rural healthcare providers.
